# Meloxicam mitigated methylglyoxal-induced glycative stress in rats

**DOI:** 10.22038/ijbms.2025.83856.18143

**Published:** 2025

**Authors:** Talha bin Fayyaz, Ghulam Abbas, Hammad Ahmed, Najeeb Khatian, Shumaila Usman, Uzair Nisar, Noor Ul Ain, Yamna Khurshid, Syed Abid Ali

**Affiliations:** 1 Department of Pharmacology, Faculty of Pharmacy and Pharmaceutical Sciences, Ziauddin University, Karachi-75600, Pakistan; 2 Department of Molecular Medicine, Ziauddin University, Karachi-75600, Pakistan; 3 Department of Pharmacology, Institute of Pharmaceutical Sciences, Jinnah Sindh Medical University, Karachi, Pakistan; 4 H.E.J. Research Institute of Chemistry, International Center for Chemical & Biological Sciences, University of Karachi, Karachi-75270, Pakistan

**Keywords:** Advanced glycation end – products, Carboxymethyllysine, Gene expression, Glycation, Meloxicam, Methylglyoxal

## Abstract

**Objective(s):**

Glycation is one of the primary underlying processes attributed to senescence and related diseases. No medicine currently targets this harmful manifestation. Drug repurposing is an efficient and cost-effective way of developing drugs. The present study evaluated meloxicam, a clinically used NSAID, for its ability to offer protection against glycative stress.

**Materials and Methods:**

Methylglyoxal (MGO; 17.25 mg/kg) was administered for two weeks to create a rat model of glycative stress. Aminoguanidine (AG; 50 mg/kg) and Meloxicam (MEL; 0.15, 0.3, and 0.6 mg/kg) were used as standard and test agents, respectively. Afterward, the cognitive (Morris Water Maze), liver (LFT), and kidney (Creatinine) functioning were evaluated. The expression of genes of interest (TNF-α, RAGE, BACE, Glyoxalase, and VEGF) were estimated (qPCR) in the liver, brain, and kidney along with histopathology (H&E staining). Carboxymethyllysine (CML) levels in rat plasma were evaluated via ELISA.

**Results:**

MEL treatment has significantly (*P*<0.05) protected the MGO-induced cognitive (duration in target quadrant, time taken to get to target quadrant, and the frequency of crossings via platform location), hepatic, and renal impairment. The qPCR data revealed that MEL prevented MGO-induced enhancement in the expression of genes of interest. Additionally, the CML levels were significantly (*P*<0.005) normalized by concomitant administration of MEL. Histopathological examination did not reveal any remarkable outcomes.

**Conclusion:**

MEL has significantly mitigated the rats’ MGO-induced cognitive, liver, and kidney impairments. Hence, it appears to be a potential molecule for repurposing as an antiglycation agent.

## Introduction

Aging, also known as senescence, is the physiological process of getting old concomitant with the emergence of various ailments (1). One of the possible underlying explanations is the glycation theory, which states that age co-relates with the burden of Advanced glycation end products, i.e., AGEs (2). They contribute to the progressive loss of body functions with time (3). AGEs are formed upon non-enzymatic interaction between carbohydrates (particularly the carbonyl group) and protein (amino acid, particularly the N-terminal of lysine and arginine’s side chain). This post-translational phenomenon is called the Maillard reaction. Glycation happens in each cell of our body; however, glycation becomes heightened in numerous pathological conditions, especially during hyperglycemia (4). They are reported to underlie cognitive decline (5), diabetes-related issues (6), kidney (7) and liver (8) abnormalities. Furthermore, AGEs interact with their receptors, known as RAGE, and cause stress, termed glycative stress (9). Methylglyoxal (MGO), alternatively known as 2-oxopropanal or pyruvaldehyde, possesses two carbonyl groups. Its formation is attributed to glycolysis, lipid metabolism, and amino acid metabolism (10). The accumulation of dicarbonyl compounds leads to heightened modification of DNA and proteins, potentially resulting in tissue and cell dysfunction and aging (11). MGO can also alter proteins, particularly lysine and arginine residues, producing AGEs (12).

**Figure 1:**
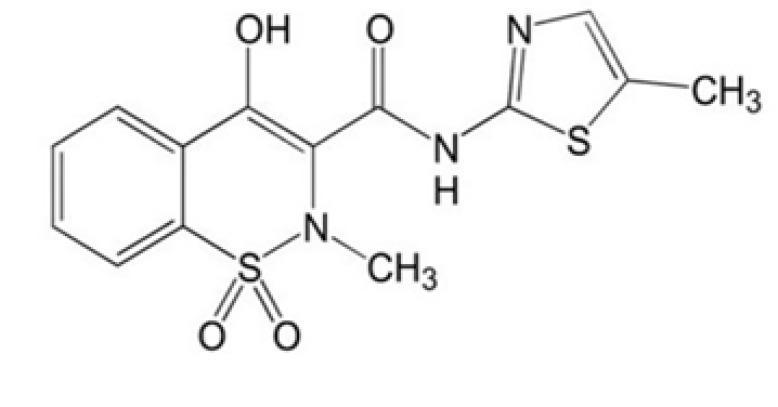
Chemical structure of meloxicam

Numerous efforts have been made to develop the inhibitors of glycation. The first AGE inhibitor that received some success was aminoguanidine, a scavenger of the di-carbonyl intermediates formed during glycation reaction (13). Unfortunately, it could also not pave the way to bedside for various reasons, particularly unpleasant outcomes. Under these conditions, the notion of repurposing enables a viable and cheap way to introduce novel compounds for the process of drug development (14). Initial screening of lead compounds for drug development initiatives relies heavily on the concept of structure-activity relationship (15). Regarding this, sulfur-containing compounds have been reported to possess anti-glycation properties, potentially exerting their effects through various mechanisms such as antioxidant activity, trapping reactive carbonyl species, and modulating pathways involved in AGE formation (16). Meloxicam is an enolic acid that belongs to the class of “oxicam .”MEL contains a thiazole ring, a five-membered aromatic heterocyclic compound with nitrogen and sulfur atoms, and a cyclic tertiary sulfonamide moiety (17, 18). Based on the aforementioned structural similarity, the current work aimed to detect and analyze MEL (Figure 1), a clinically used NSAID (19), for its potential utility as an anti-glycation drug. 

## Materials and Methods

### Animals

Wistar rats (150–200 g) were purchased from the Animal Resource Facility of the International Centre for Chemical and Biological Sciences, University of Karachi. They were housed in standard conditions, i.e., 12-hour cycles of darkness and light, and a constant temperature of 25±1 °C. The rats were housed in recommended cages, given free access to pelleted rat chow, and cleaned tap water *ad libitum*. Approval for all experiments was granted by the Institution’s Animal Ethics Committee (Approval No: 2021–007/MM). Additionally, the principles outlined in the “Guide for the Care and Use of Laboratory Animals” published by the National Research Council (NRC) were followed during the entire study.

### Chemicals

Formalin, methylglyoxal, and sodium chloride were purchased from Sigma-Aldrich (Germany). Meloxicam was a gift from Hilton Pharma Ltd (Pakistan). 

### Grouping of animals

Wistar rats were randomized into six groups of 3 rats:

Group 1: (Vehicle control): Normal saline 0.9% (5 ml/kg, *IP*) for 14 days.

Group 2: (Model Group): Methylglyoxal (MGO, 17.25 mg/kg, *IP*) for 14 days (20).

Group 3: (Positive Control): Aminoguanidine (AG, 50 mg/kg, *IP*) was administered 30 min before MGO administration for 14 days.

Group 4, 5, and 6: (Treatment Groups): Meloxicam (MEL; 0.15 mg/kg, 0.3 mg/kg, or 0.6 mg/kg, *IP*) was administered 30 min before MGO injection for 14 days.

It is of note that the present work is part of a bigger project whereby multiple drugs were tested for their ability to be repurposed against glycation. A part of this work was published earlier which contains the data on vehicle and positive control (20), which is also used in this manuscript.

After 14 days, the following tests were performed:

### Assessment of cognitive performance

The Morris Water Maze (MWM) experiment was used to evaluate spatial memory development (21). The MWM pool (black color), with a diameter of 180 cm and a height of 50 cm, was partitioned into four equal sections (quadrants). A disc-shaped platform (black color) was positioned within one of these sections for escape purposes. This platform’s location remained fixed throughout the experiment. The pool was filled with water and kept at 25 ± 2 °C. Distinct cues were strategically positioned along the pool’s walls to aid the animals in locating the platform. Initially, the platform rested 1 cm above the water’s surface during the familiarization session on day one. Four trials, each lasting 120 sec, were conducted to train the animals in this session. During these sessions, rats were placed in one of the pool’s quadrants facing the wall. If an animal found the platform within the allotted time, it could only remain on the elevated surface for 5 sec; alternatively, it was gradually led to the platform and permitted to stay about 30 sec. In subsequent training (acquisition) sessions conducted over the next three days, the platform was gradually submerged below the water’s surface, following the same procedure as day one, with five trials for each animal. On the fifth day, the platform was removed from the pool to assess spatial memory retention to perform a probe trial. The animals had 120 sec to locate the platform. A video camera mounted above the MWM recorded the experiment. Various parameters were analyzed to evaluate spatial memory retention, including latency to reach the target quadrant, duration spent in the target quadrant, and the number of crossings across the platform’s prior position.

### Assessment of renal and hepatic functions

After collecting blood via cardiac puncture, it was permitted to coagulate at room temperature for 15 min. Later, the serum was obtained by centrifugation at 4250×g (or about 3500 RPM) for 20 min. For renal assessment, creatinine was measured, while for hepatic assessment, SGPT, SGOT, ALP, direct, and total bilirubin levels were measured via a clinical chemistry analyzer (AU5800, Beckman Coulter, Germany).

### Expression Study

The gene expression was assessed using RT-qPCR (quantitative Polymerase Chain Reaction). The genes of interest from the liver were RAGE (receptor for advanced glycation end products), TNF-α (tumor necrosis factor-alpha), and Glyoxalase-1. From the kidney, genes of interest were RAGE, TNF-α, and VEGF (vascular endothelial growth factors), while TNF-α, RAGE, and BACE (beta-secretase) were from the brain. Glyceraldehyde-3-phosphate dehydrogenase (GAPDH) was used as a reference housekeeping gene. Total RNA from the liver, kidney, and brain was extracted with Trizol reagent and then converted to cDNA (cDNA Synthesis Kit, Thermo Scientific). The reaction mixture (25 µl) underwent thermal cycling in three steps, i.e., denaturation for 10 min at 95 °C, followed by a second denaturation for 15 sec at 95 °C, annealing for 30 sec at 60 °C, and a final extension for 30 sec at 72 °C. These denaturation, annealing, and extension steps were repeated for 40 cycles. The Thermo Scientific Maxima SYBR Green/ROX qPCR master mix (2X) kit was utilized with the respective primers (Table 1). Gene expression variation between control and treated groups was determined using the delta-delta cycle threshold (Ct) method (22).

### Histology

The liver, brain, and kidney were extracted, cut, and preserved in 10% formalin. Following dehydration in higher ethanol concentrations, the organs were cleaned in xylene and embedded in paraffin. Thin 4 µm slices were prepared and stained with H&E (Haematoxylin & Eosin). A bright field microscope was used to analyze structural and morphological alterations in identified cells from each tissue (Olympus U-MDOB3, Japan).

### CML Quantification

The serum obtained was also used to estimate CML using an ELISA kit (CEB977Ge 96 Tests, Cloud Clone Corp, China) following the manufacturer protocol. The test specimens were placed on a plate frame, and 50 μl was applied to each well. A membrane was placed over the plate and then maintained for 30 min. After the incubation, wells were washed thoroughly 3-4 times using a multichannel pipette, ensuring no well contamination, and then allowed to dry. Later, horseradish peroxidase (HRP, 50 μl) conjugate was applied to every well (except the blanks) and incubated for 30 min. Afterward, wells were rewashed, and chromogen solutions A and B (50 μl each) were added. The membrane was firmly sealed, and the plate was incubated for an additional 15 min. Finally, a stop solution was introduced, changing the color from blue to yellow. Absorbance readings were taken immediately at 450 nm via a spectrophotometer (Biotek Synergy HTX Multi-mode reader, Japan).

### Statistical analysis

The data is provided as mean ± SEM (n=3 per group). For comparing groups statistically, one-way ANOVA was utilized, accompanied by *post-hoc* analysis (Least Significant Difference, LSD). The statistical analysis was performed using IBM SPSS software (version 21.0, USA). *P*<0.05 serves as the minimal level of significance.

## Results

### Evaluation of cognitive function

MGO-treated rats demonstrated a significant enhancement in escape latency time and time to reach the target quadrant, whereas a significant reduction in the time spent in the target quadrant and number of crossings through platform position was observed in comparison with control rats (Table 2). A vice-versa effect was noted in AG and MEL treatment rats compared to the MGO group. Among the various tested doses (0.15 mg/kg, 0.3 mg/kg, and 0.6 mg/kg), the lowest showed the most prominent effect. 

**Figure 2:**
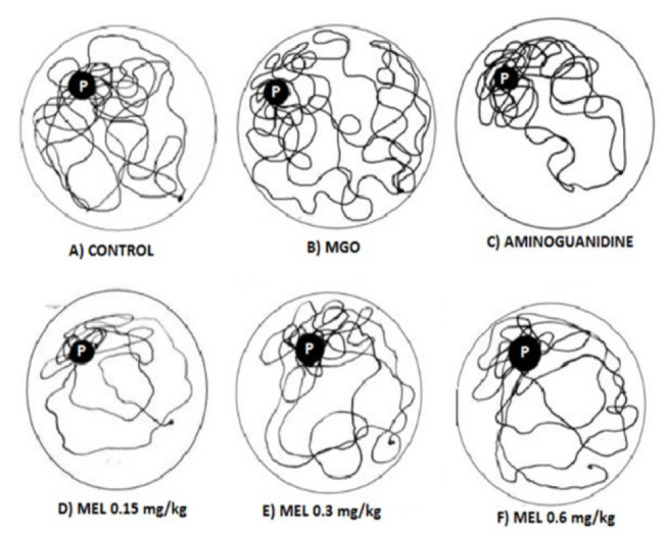
Navigation Mapping in the Morris Water Maze

The navigation patterns revealed the movement of control rats in the platform quadrant, while rats in the model group displayed erratic movement in the MWM pool (Figure 2). In contrast, the treatment groups (Aminoguanidine and meloxicam-treated rats) exhibited a pattern in their navigation plots more similar to that of the control.

### Assessment of renal and hepatic function

The concentrations of creatinine, ALP, SGPT, and SGOT in the Model group (MGO) exhibited a significant elevation compared to the control group (Table 3). In contrast, both AG and MEL (0.15 mg/kg, 0.3 mg/kg, and 0.6 mg/kg) demonstrated a notable decrease in the amounts of markers in contrast to MGO.

### Quantitative polymerase chain reaction (qPCR)

The qPCR of various samples are as follows:

***Liver***

**Figure 3:**
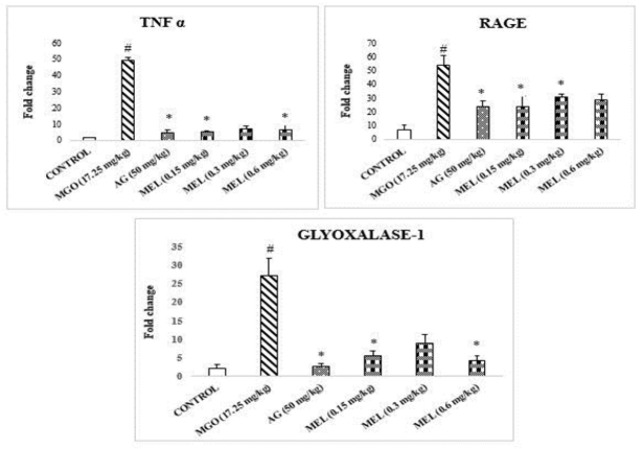
Expression of genes in liver tissue of rats

The RT-qPCR analysis exhibited a significant increase in the fold change of RAGE, TNF-α, and Glyoxalase-I genes in methylglyoxal-administered livers compared with control samples (Figure 3). On the contrary, significant reductions in the expression of these genes were found in AG and MEL-treated rats.

***Kidney***

**Figure 4:**
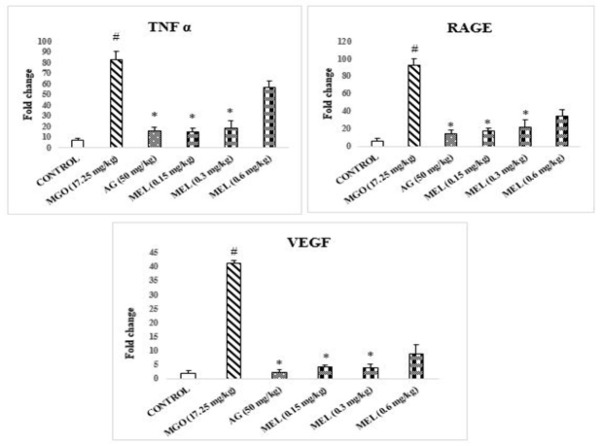
Expression of genes in Kidney tissue of rats

TNF-α, RAGE, and VEGF gene expression exhibit a significant increase compared to control rats (Figure 4). However, expression of these genes appears to be decreased in AG and MEL-administered rats compared to MGO rats.

***Brain***

**Figure 5:**
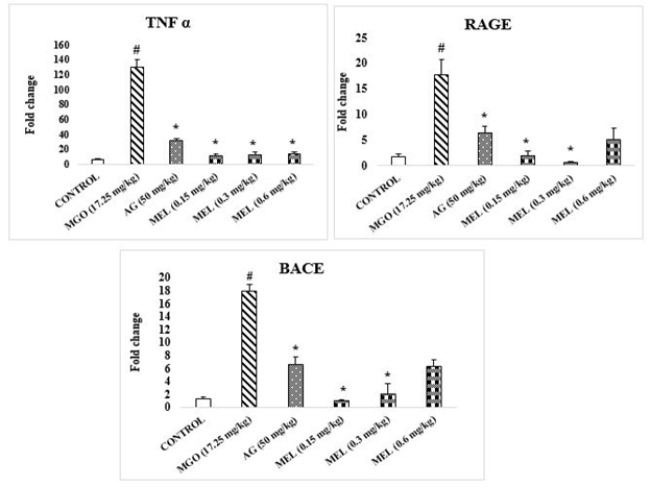
Expression of genes in Brain tissue of rats

The concentrations of RAGE, TNF-α, and BACE genes were significantly elevated in the brains of MGO-injected rats compared to the control group (Figure 5). However, their expression showed a significant decline in AG and MEL-treated brains compared to the MGO group. 

### Histopathological study

**Figure 6:**
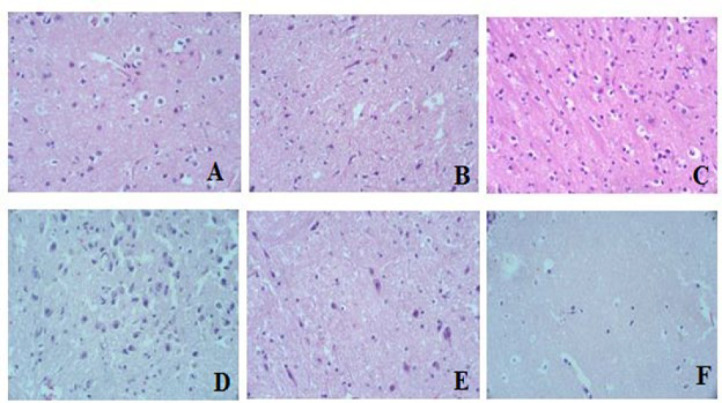
Histology of Brain tissue of rats

**Figure 7:**
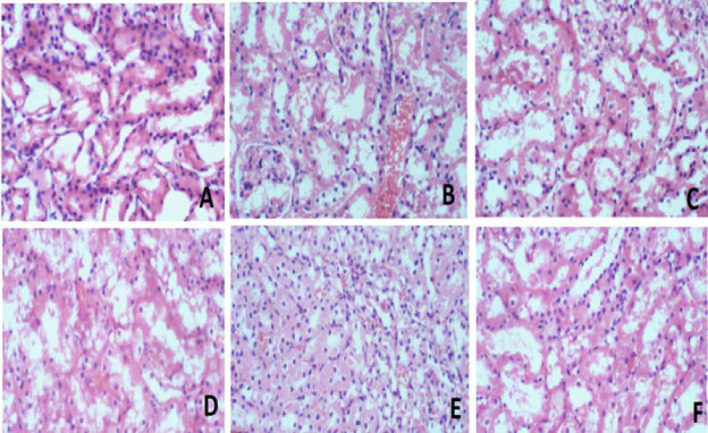
Histology of Kidney tissue of rats

**Figure 8:**
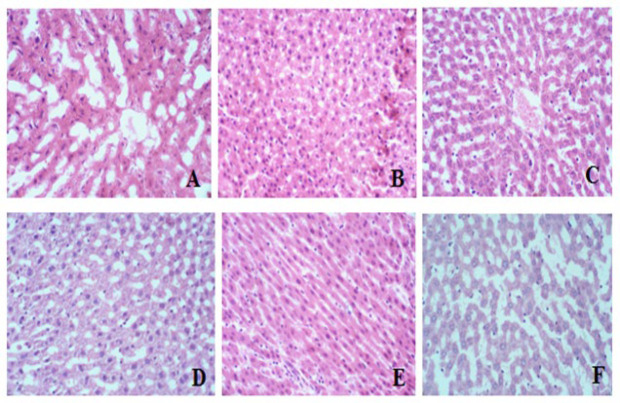
Histology of Liver tissue of rats

The histological examination of brain, kidney, and liver tissue revealed no discernible pathological alterations in any of the treatment groups compared to the control (Figures 6, 7, and 8, respectively). 

### CML Quantification

**Figure 9.**
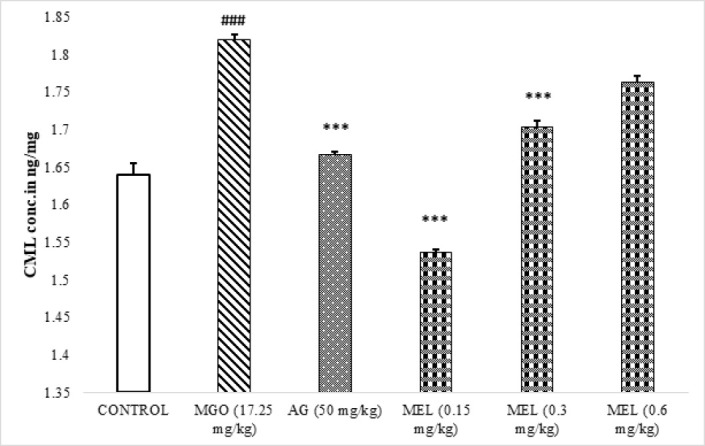
Effects of meloxicam on CML levels in the serum of rats

The results of CML quantification unveiled significantly (*P*<0.005) elevated CML levels in the serum of rats in the model group in comparison with the control (Figure 9). The AG and MEL-treated serums demonstrated a significant reduction in the levels of CML in comparison with MGO-treated rats.

## Discussion

Glycation is one of the pathogenic pathways implicated in aging and related morbidities. Unfortunately, no medicine exists to fight this harmful anomaly. Repurposing is a quick and cost-effective way to introduce candidate compounds into drug research process. Having this in view, the current investigation was aimed to explore the influence of meloxicam, a clinically used NSAID, on glycative stress.

MGO, a di-carbonyl compound, has been reported to modify DNA and proteins, resulting in tissue and cell dysfunction, diseases, and aging (23). It can alter proteins, particularly their lysine and arginine residues, producing AGEs (12). Hence, it induced accelerated aging in rats *via* enhancing glycative stress (24). Morris water maze experiment is an effective tool for evaluating cognitive abilities and spatial memorization in rodents (25). Our findings revealed that MGO-treated rats showed severe deficits in visual learning and memory, as shown by higher latency in getting to the target quadrant, less time stayed in the target quadrant, and lowered frequency of crossings in contrast to the control group (Table 2). A similar effect of MGO on cognition was reported earlier, which authenticates our experimental setup (26). Both AG and MEL, especially their lower tested doses (0.15 and 0.3 mg/kg), have mitigated this MGO-induced cognitive decline. The literature revealed that MEL might display concentration-independent behavior. At lower concentrations, it was found to be more effective in certain studies than in higher doses (27-29). The most probable explanation is that excessive drug molecules may compete for binding or self-associate at higher doses, potentially reducing their availability to interact with reactive carbonyl species efficiently, thus attenuating the anti-glycation activity. However, further study is required to delineate this unusual outcome. Furthermore, our data involving navigation maps also exhibited erratic movement in MGO-treated rats, as observed earlier (30). However, like control rats, the treatment groups maintained more focused navigation in the platform quadrant (Figure 2). This indicates enhanced memory retention, suggesting its utility as a therapeutic intervention for cognitive dysfunction associated with glycative stress. 

The renal and hepatic function was also altered in MGO-treated rats as the serum levels of creatinine, ALP, SGOT, and SGPT were higher in comparison with control (Table 3). A similar effect of MGO in serum biomarkers of renal and hepatic function was reported earlier following the administration of MGO, thereby validating our experimental setup (31). It is of note that the AG and MEL, especially lower tested doses, were found to protect the renal and hepatic function against the harmful effects of MGO. 

In our experimental design, along with the biochemical, the expression of certain pathologically important genes from the liver, kidney, and brain were also quantified to explore the molecular changes in these tissues. Our data reveals a considerable elevation in the expression of all genes of interest, i.e., RAGE, TNF α, and Glyoxalase-1 in MGO-exposed hepatic tissues compared to control (Figure 3). This indicates a heightened glycation stress in the liver. A similar effect of MGO in hepatic tissues was reported earlier (32, 33). The search of the literature revealed that RAGE activation triggers phosphorylation of ERK1/2, PI3-K/AKT, JAK2, and RhoGTPases, culminating in NF-κB stimulation and pro-inflammatory cytokine production, such as TNF-α (32, 34). Conversely, the concomitant treatment with AG and MEL has significantly attenuated the aforementioned up-regulation of genes. 

The VEGF expression was also assessed in the case of the kidney, along with TNF-α and RAGE. A literature search revealed that MGO triggers the synthesis of VEGF in endothelial and mesothelial cells, consequently fostering angiogenesis (35). Our investigation found considerable TNF-α, RAGE, and VEGF elevation in MGO-administered rats’ kidneys comparable to control rats (Figure 4). In conformity with AG, Meloxicam (MEL) administration decreased TNF-α, RAGE, and VEGF levels in the kidneys, comparable to the MGO-administered group, highlighting its potential as a therapeutic agent against MGO-induced renal injury. 

In the case of MGO-treated brains, substantial up-regulation of RAGE, TNF α, and BACE in contrast to the control group was noted (Figure 5). In conformity, BACE activity was earlier associated with MGO-induced neurodegenerative processes (36). Conversely, simultaneous treatment with AG and MEL resulted in significant reductions in the fold change of RAGE, TNF α, and BACE expression in comparison with the MGO group, thereby underscoring their efficacy in ameliorating MGO-induced neuroinflammation and β-amyloid production. 

Our histopathological evaluation did not reveal any structural changes in the model group’s brain, kidney, and liver compared to the control (Figures 6, 7, and 8). The lack of detectable pathological changes suggests that the duration of exposure may have been insufficient to produce significant histological alterations (37). Histological transformations typically require more time to manifest (38). However, the absence of overt histological abnormalities does not necessarily imply the absence of underlying physiological or biochemical alterations.

Carboxymethyllysine (CML) is one of the major AGEs that was reported to co-relate with the aging process and associated morbidities (39, 40). As a result, its concentration in serum was determined. Our data shows the significantly raised CML in the serum of MGO-treated rats (Figure 9). This advocates the role of CML in mediating the cognitive, hepatic, and renal impairment observed in the present study. In contrast, both AG and MEL administrations have significantly hampered the MGO-induced increase in CML levels. This supports the notion that the protective effect shown by AG and MEL in the present study was most likely because of their ability to inhibit glycation.

**Table 1:** List of primer sequences of genes of interest in rats

GENE	PRIMER SEQUENCE	
BACE	F	AGGGCTACTATGTGGAGATG
	R	CATACACAGACTTTCGGAGG
RAGE	F	AGTCCGAGTCTACCAGATTC
	R	TCTCCTCCTTCACAACTGTC
GLYOXALASE-1	F	CAAGATCCTGATGGCTACTG
	R	CAGAATGGCTTGAACTGGAG
VEGF	F	GGAGTACCCCGATGAGATA
	R	TCATCTCTCCTATGTGCTGG
TNF-α	F	CACGCTCTTCTGTCTACTG
	R	CTGCTTGGTGGTTTGCTA
GAPDH	F	GGATGGAATTGTGAGGGAGA
	R	GTGGACCTCATGGCCTACAT

The Figure depicts the movement pattern in the MWM pool during the probe trial. The MGO (Methylglyoxal) alone rats showed random movements in the pool, while in similarity with control rats, the AG (Aminoguanidine) and MEL (Meloxicam) treated rats' movements were more confined around the platform position (P).

**Table 2:** Effect of meloxicam treatment on learning and memory indicators in Morris Water Maze (MWM) in rats

Groups	Time to reach target quadrant (sec)	Time spent in target quadrant (sec)	Crossings through platform position (Count)
Control	9 ± 2	43 ± 3	8 ± 1
Model group	20 ± 1 ^#^	25 ± 2 ^##^	5 ± 0.3 ^#^
AG (50 mg/kg)	3 ± 1 ***	72 ± 6 ***	17 ± 2 ***
MEL (0.15 mg/kg)	4 ± 1 ***	56 ± 1 ***	10 ± 0.3 **
MEL (0.3 mg/kg)	3 ± 1 ***	37 ± 4 **	9 ± 1 *
MEL (0.6 mg/kg)	4 ± 0.3 ***	34 ± 3 *	10 ± 1 *

# (*P*<0.05), ## (*P*<0.01), and ### (*P*<0.005) signify statistical evaluation with control

* (*P*<0.05), ** (*P*<0.01), and *** (*P*<0.005) as evaluated against MGO group.

MGO: Methylglyoxal; AG: Aminoguanidine; MEL: Meloxicam

**Table 3:** Effect of meloxicam on renal and hepatic function indicators of methylglyoxal-treated rats

Treatment groups	Renal function	Hepatic function				
	Creatinine	Bilirubin	Direct bilirubin	ALP	SGPT	SGOT
Control	0.7 ± 0.02	0.6 ± 0.06	0.14 ± 0.02	177 ± 2	23 ± 2	19 ± 2
MGO	0.9 ± 0.06 ^#^	0.7 ± 0.07	0.16 ± 0.01	218 ± 5 ^#^	30 ± 1 ^#^	24 ± 1 ^#^
AG (50 mg/kg)	0.6 ± 0.01*	0.7 ± 0.03	0.15 ± 0.02	190 ± 5*	23 ± 1*	18 ± 1*
MEL (0.15 mg/kg)	0.6 ± 0.01**	0.6 ± 0.01	0.13 ± 0.01	162 ± 3**	19 ± 1**	14 ± 1**
MEL (0.3 mg/kg)	0.6 ± 0.04*	0.6 ± 0.04	0.14 ± 0.00	169 ± 4 **	20 ± 2**	17 ± 1*
MEL (0.6 mg/kg)	0.6 ± 0.05*	0.6 ± 0.05	0.14 ± 0.01	197 ± 3*	22 ± 3*	17 ± 2*

MGO: Methylglyoxal; AG: Aminoguanidine; MEL: Meloxicam

The graphs present the mean ± SEM values (n=3) of the fold change in gene expression in liver samples. The TNF-α, RAGE, and Glyoxalase-1 expression intensities were substantially elevated in MGO-treated rats compared to the control group. However, treatment with AG (Aminoguanidine) and MEL (Meloxicam) led to a substantial reduction in the transcription of these genes relative to the MGO-treated group (Methylglyoxal). A hash (#) denotes a significant (P<0.05) change in contrast to the control group, whereas asterisks [* (*P*<0.05), ** (*P*<0.01), and *** (*P*<0.005)] signify statistical differences with the MGO group.

The illustration presents the mean ± SEM values (n=3) of the change in expression levels of the target genes (TNF-α, RAGE, and VEGF) in kidney samples. Methylglyoxal-administered rats exhibited a significant up-regulation of these genes with reference to the control group. Notably, the rats treated with AG (Aminoguanidine) and MEL (Meloxicam) demonstrated a substantial reduction in gene expression levels relative to the MGO (Methylglyoxal) group. A hash (#) denotes a significant (*P*<0.05) change in contrast to the control group, whereas asterisks [* (*P*<0.05), ** (*P*<0.01), and *** (*P*<0.005)] signify statistical differences with the MGO group.

The figure illustrates the mean ± SEM (n=3) for the change in expression levels of the target genes (TNF-α, RAGE, and BACE) in brain samples. Methylglyoxal-administered rats exhibited a significant up-regulation in the transcription of these genes compared to the control. In contrast, treatment with AG (Aminoguanidine) and MEL (Meloxicam) resulted in a noticeable decrease in the levels of these genes relative to the MGO group (Methylglyoxal). A hash (#) denotes a significant (*P*<0.05) change in contrast to the control group, whereas asterisks [* (*P*<0.05), ** (*P*<0.01), and *** (*P*<0.005)] signify statistical differences with the MGO group.

The figure depicts images of brain tissue stained with H&E. No significant microscopic changes were noted in any of the groups: "A" represents the control group, "B" represents the Model group, "C" represents the AG group, "D" represents MEL (0.15 mg/kg), "E" represents MEL (0.3 mg/kg) and "F" represents MEL (0.6 mg/kg). MGO (Methylglyoxal), AG (Aminoguanidine), and MEL (Meloxicam).

The figure depicts the images of kidney tissue stained with H&E. No significant microscopic changes were noted in any of the groups: "A" represents the control group, "B" represents the Model group, "C" represents the AG group, "D" represents MEL (0.15 mg/kg), "E" represents MEL (0.3 mg/kg), and "F" represent MEL (0.6 mg/kg). MGO (Methylglyoxal), AG (Aminoguanidine), and MEL (Meloxicam).

The figure depicts the images of liver tissue stained with H&E. No significant microscopic changes were noted in any of the groups, i.e., "A" represents the control group, “B” represents the Model group, “C” represents the AG group, “D” represents MEL (0.15 mg/kg), “E” represents MEL (0.3 mg/kg) and “F” represents MEL (0.6 mg/kg). MGO: Methylglyoxal; AG: Aminoguanidine; MEL: Meloxicam.

The result displayed the mean ± SEM of CML concentration (n=3). The methylglyoxal exposed serum demonstrated a substantial increase in CML concentration compared to control. However, its levels were found to be decreased in AG (Aminoguanidine) and MEL (Meloxicam) administered animals in comparison with MGO (Methylglyoxal) group serum. The "#" and "*" symbols represent statistical comparison with control and MGO groups. The presence of single, double, and triple symbols shows the degree of significance (*P*<0.05, P<0.01, and *P*<0.005, respectively).

## Conclusion

Our study demonstrates that meloxicam, a clinically used NSAID, mitigated rats’ MGO-induced cognitive, renal, and hepatic impairments. Hence, it is a potential lead molecule for repurposing as an anti-glycation agent.

## Data Availability

The data supporting the findings of this study can be obtained from the corresponding author upon reasonable request.

## Declaration

We have not used any AI tools or technologies to prepare this manuscript.
